# Investigating the Role of the Melanocortin-1 Receptor Gene in an Extreme Case of Microgeographical Variation in the Pattern of Melanin-Based Plumage Pigmentation

**DOI:** 10.1371/journal.pone.0050906

**Published:** 2012-12-05

**Authors:** Yann X. C. Bourgeois, Joris A. M. Bertrand, Christophe Thébaud, Borja Milá

**Affiliations:** 1 Laboratoire Évolution et Diversité Biologique, UMR5174 CNRS - Université Paul Sabatier – ENFA, Toulouse, France; 2 Museo Nacional de Ciencias Naturales, Consejo Superior de Investigaciones Científicas (CSIC), Madrid, Spain; Institut de Biologia Evolutiva - Universitat Pompeu Fabra, Spain

## Abstract

The Réunion grey white-eye (*Zosterops borbonicus*) is a single-island endemic passerine bird that exhibits striking geographically structured melanic polymorphism at a very small spatial scale. We investigated the genetic basis of this color polymorphism by testing whether the melanocortin-1 receptor (*MC1R*), a gene often involved in natural melanic polymorphism in birds, was associated with the observed plumage variation. Although we found three non-synonymous mutations, we detected no association between *MC1R* variants and color morphs, and the main amino-acid variant found in the Réunion grey white-eye was also present at high frequency in the Mauritius grey white-eye (Z*osterops mauritianus*), its sister species which shows no melanic polymorphism. In addition, neutrality tests and analysis of population structure did not reveal any obvious pattern of positive or balancing selection acting on *MC1R*. Altogether these results indicate that *MC1R* does not play a role in explaining the melanic variation observed in the Réunion grey white-eye. We propose that other genes such as *POMC*, *Agouti* or any other genes involved in pigment synthesis will need to be investigated in future studies if we are to understand how selection shapes complex patterns of melanin-based plumage pigmentation.

**Trial Registration:**

All sequences submitted to Genbank. Accession number: JX914505 to JX914564.

## Introduction

The genetic basis and origin of color polymorphism in natural populations is a classic theme in our understanding of ultimate and proximate causes of phenotypic variation and evolution [1]. In vertebrates, the study of melanic coloration has led to the characterization of important target genes that may underlie phenotypic variation and divergence in natural populations [2], [3]. One recurrent result emerging from most studies is the involvement of the melanocortin-1 receptor (*MC1R)* coding region in explaining variation in melanism, sometimes showing shared mutations due to convergent evolution between distantly related species [4]. In birds, associations between dark coloration and mutations in *MC1R* have been highlighted in a number of wild species (Table 1), including snow geese [5], fairy-wrens [6], bananaquits [7], swans [8], falcons [9], *Acrocephalus* warblers [10] and *Monarcha* flycatchers [11]. *MC1R* has been shown to play an important role in a variety of processes such as sexual selection [5], [11], [12], crypsis [13] and possibly immunity [9] although it is generally considered to have few pleiotropic effects [2]. While most studies have focused on functional substitutions in *MC1R* coding region in species displaying discrete color dimorphism, few have tried to examine amino acid variation in species with diverse melanin-based patterns of plumage pigmentation (but see [12], [14]). For instance, in studies of the blue-crowned manakin (*Lepidothrix coronata*) [15] which displays a gradation in melanic coloration according to geography, or the Old World leaf warblers (*Phylloscopus sp.*) in which there is interspecific variation in unmelanized plumage pattern elements [14], no association between the degree of melanism and nucleotide variation at *MC1R* could be found.

**Table 1:** Summary of major patterns of melanic variation in birds and their link with *MC1R*.

Mutation	Phenotype	Species studied	References
**Glu^92^→Lys^92^**	Extensive black (black plumage). Less marked in quail. Dominant.	Chicken (*Gallus gallus*) Japanese quail (*Coturnix japonica*) Bananaquit (*Coereba flaveola*) Tahiti Reed Warbler (*Acrocephalus caffer*)	[7], [10], [31], [39]
**Ala^16^→ Thr^16^, Ile^38^→Asn^38^, Ile^111^→Val^111^, Gln^157^→Arg^157^, Val^166^→Ile^166^**	Ala^16^→ Thr^16^, Ile^38^→Asn^38^, Ile^111^→Val^111^, Gln^157^→Arg^157^, found associated with the mainland (blue) phenotype. Val^166^→Ile^166^ found associated with melanic phenotypeand is dominant.	White-winged Fairywren (*Malurus leucopterus*)	[6]
**Val^85^→Met^85^**	Different amounts of grey or brown (heterozygous) to completely dark (homozygous).	Lesser snow goose (*Chen c. caerulescens)*, Red-footed boobies (*Sula sula*)	[5], [40]
**Glu^100^→Lys^100^**	Associated with neck melanism.Found withGlu92→Lys92.	Black-necked Swan *(Cygnus melanocoryphus)*	[8]
**Deletion 114-117**	Dark plumage. Dominant.	Eleonora’s Falcon (*Falco eleonorae*)	[9]
**Asp^119^→Asn^119^**	Black plumage. Dominant.	Chestnut-bellied Monarch from Ugi island (*Monarcha castaneiventris*)	[11]
**His^215^→Pro^215^**	Alteration of light stripes on back and dorsal head. Associated with Glu92→Lys92. Recessive.	Chicken (*Gallus gallus*)	[31]
**Arg^230^→His^230^**	Grey (heterozygous) to black (homozygous) plumages.No melanism in *Coscoroba coscoroba.*	Arctic skua (*Stercorarius parasiticus*), Black Swan *(Cygnus atratus*), Coscoroba Swan (*Coscoroba coscoroba*)	[5], [8]
**Deletion 256**	Causes melanism. Wild allele is dominant.	Guinea fowl (*Numida meleagris*)	[41]
**No mutation linked to phenotype**	Black plumage.	Chestnut-bellied Monarch from Three Sisters Islands (*Monarcha castaneiventris*)	[11]
**No mutation linked to phenotype**	Geographically structured gradation from green to black plumage.	Blue-crowned manakin (*Lepidothrix coronata*)	[15]
**No mutation linked to phenotype**	Variation in melanization in wing bars, crown stripe and rump patches.	Old World leaf warblers (genus *Phylloscopus*)	[14]
**No mutation linked to phenotype**	Variation in the extent of phaeomelanin depositionacross the body.	Réunion Grey White-Eye (*Zosterops borbonicus)*	This study

In this study, we assess whether *MC1R* could explain variation in melanistic patterns in the Réunion grey white-eye, *Zosterops borbonicus*, a species composed of four distinct plumage morphs on the topographically and ecologically complex island of Réunion (Mascarene archipelago). This species provides an excellent system because its prominent plumage color polymorphism stands in stark contrast to the single morph found in its sister species, *Z. mauritianus* [16], [17] and variation in plumage color among morphs, while conspicuous, is relatively complex in terms of melanin pigmentation patterns, with a completely brown morph, a completely grey morph, a grey-headed brown morph, and a grey-headed brown morph with a brown nape. The morphs occupy discrete geographic entities, with the exception of the brown and grey morphs that are completely sympatric at high altitudes (see [16], [17] for details). Hybrid zones arise where morphs come into contact, as happens between parapatric morphs. In contrast, there appears to be no assortative mating with regards to morph color in the area of sympatry between grey and brown morphs (unpublished data). Patterns of coloration among morphs are stable over time, with no sex effect [17]. Brown parts involve deposition of phaeomelanin in feather barbs and eumelanin deposition in barbules, while grey parts involve low deposition of phaeomelanin [17].

Although to date *MC1R* has not been associated with phaeomelanin variation in the presence of eumelanin, its central position in controlling the production of both eumelanin and phaeomelanin [18] makes it a relevant candidate in explaining at least partly this plumage color polymorphism.

The main aims of this study are to ask whether there is an association between mutations in *MC1R* and color variation in *Z.borbonicus*. First, we examined nucleotide variation in the coding region of *MC1R* and assessed whether mutations were associated with patterns of variation in melanin pigmentation. Secondly, we investigated whether natural selection could have shaped the pattern of nucleotide variation among morphs. Third, we asked whether sequence variation in *MC1R* coding region could be due to hitch-hiking to positively selected cis-regulatory mutations by examining whether color morph was associated with patterns of genetic differentiation.

## Methods

### Sampling

**Figure 1:**
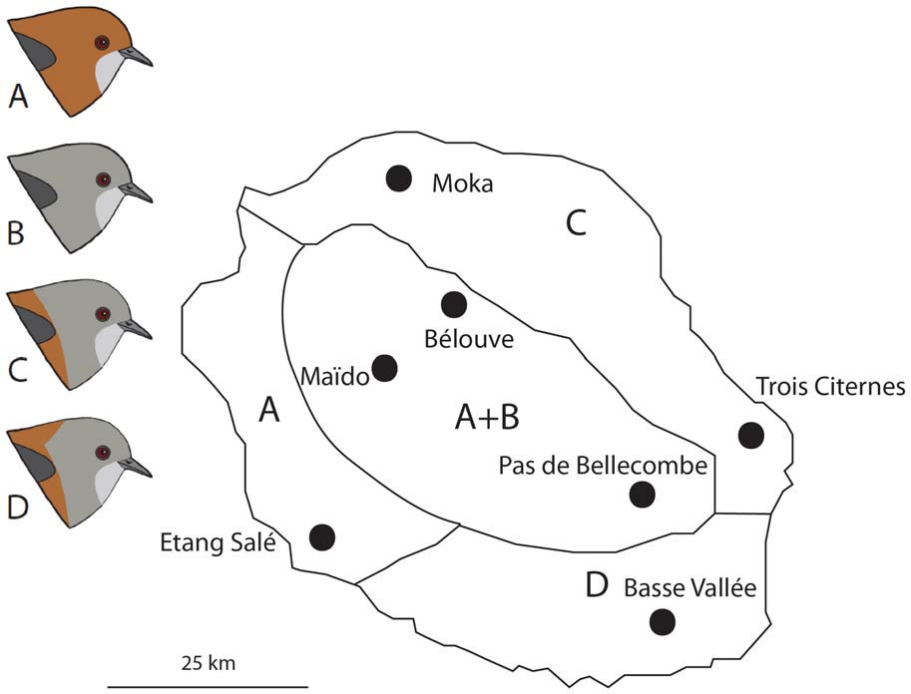
Map showing *Z. borbonicus* sampling localities, and distribution of the four morphs on Réunion. Letters correspond to the different plumage morphs: A: Brown morph; B: Grey morph; C: Grey-headed brown morph; and D: Grey-headed brown-naped brown morph. For a more detailed description of pigmentation phenotypes, see [17]. Adapted from [18].

Blood samples used for DNA extraction were collected during field trips at different locations on the islands of Réunion (55°39′E; 21°00′S) and Mauritius (57°33′ E; 20°17′ S) between 2007 and 2009 (Figure 1, Table 2). Birds were captured using mistnets and approximately 10 µL of blood were collected from each bird. Blood was conserved in Queen’s lysis buffer [19] and stored at −20°C for long-term preservation. Morphs were identified by eye in the field, and visual assignments were further confirmed in the laboratory by using pictures taken in the field and on the basis of previous reflectance analysis [20]. With respect to the brown morph, reflectance studies suggested that highland (>1,500 meters high) and lowland forms are distinguishable in terms of coloration, so we analyzed these populations separately. We analyzed a total of 51 individuals from Réunion, including five brown individuals from lowland localities, 15 brown individuals from three highland localities, 13 grey individuals from two localities, eight grey-headed brown individuals from two localities and 10 grey-headed brown-naped brown individuals from one locality (Table 2). For comparison purposes, we also included nine individuals of *Z. mauritianus* in our analyses.

**Table 2:** Localities and number of birds sampled on the islands of Réunion and Mauritius.

Locality/Morph	Sample size
**Réunion**	**51**
**Brown**	**20**
Bélouve	5
Maïdo	4
Pas de Bellecombe	6
Etang Salé	5
**Grey**	**13**
Maïdo	4
Pas de Bellecombe	9
**Grey-headed brown**	**8**
Moka	5
Forêt Mourouvin	3
**Grey-headed brown-naped brown**	**10**
Basse Vallée	10
**Mauritius**	**9**

### DNA Extraction and Amplification

DNA was extracted using a Qiagen® kit, following the manufacturer’s instructions for nucleated blood cells. We amplified a 817-bp fragment of the *MC1R* coding region, including all sites previously shown to be associated with plumage color change in birds, following [15] for conditions and primers.

Reactions were performed using: 5 µL of 5X buffer (Promega®), 0.5 µL 10 mM dNTPs, 0.125 µL of Taq (5 u/µL, Promega GoTaq® DNA polymerase), 1 µL of each primer (10 µM), 15.4 µL of sterile distilled water, and 2 µL of DNA (∼30 ng of template DNA), totaling 25 µL. The thermocycling profile was as follows: an initial denaturation at 94°C for 60 s, then 40 cycles consisting of a 45-s 94°C denaturation step, a 45-s 62°C annealing step, and a 60-s extension step at 72°C. A final elongation step at 72°C for 5 minutes ended the process. PCR products were visualized on 1% agarose gels. DNA was sequenced in both directions using a 96-well capillary sequencer 3730XL (Applied Biosystems ®) and the same primer pairs used for PCR reactions.

### *MC1R* Sequence Analysis

Sequences were checked and aligned unambiguously by eye. MEGA 5 [21] was used to translate nucleotide sequences to amino-acid sequences. To guard against amplification of pseudogenes, the absence of misplaced stop codons and frame shift mutations was verified for all sequences. We aligned the sequences obtained with *MC1R* cDNA from chicken (*Gallus gallus*, Genbank accession number: AY220305) and Zebra finch (*Taeniopygia guttata*, Ensembl accession number: ENSTGUG00000008024) to detect potential variants at sites previously identified as being associated with melanic variation in other bird species. We noted double peaks at single sites that were approximately half the height of neighboring peaks. Individuals were considered as heterozygous if these double peaks were observed in both strands. To visualize the relationship among haplotypes we constructed a haplotype network using the Network software (http://www.fluxus-engineering.com/sharenet.htm).

### Tests for Molecular Signatures of Selection

Despite having relatively similar effects on sequence polymorphism, demography and selection can be distinguished to varying degrees with five of the tests we employed: Tajima’s D [22], Fu’s F_s_ [23], Fay and Wu’s H [24] and Fu and Li’s D* and F* [25]. Fu and Li’s D* and F* focus on rare alleles and are useful in detecting positive selection in a context of low sequence diversity. Fu’s F_s_ and Tajima’s D are classical tests of selection focusing either on the distribution of haplotype frequencies relative to neutral expectations (Fu’s F_s_) or on the difference between the number of segregating sites and the average number of nucleotide differences (Tajima’s D). We also calculated Fay and Wu’s H, which compares genealogies between and within species and is often presented as less sensitive to demographic events than other tests [24] but see [26]). In the case where positive selection acts on one or several morphs, negative values should be obtained for these tests, especially for tests supposedly more impacted by selection such as Tajima’s D or Fay and Wu’s H [24], [26], [27]. If balancing selection occurs, these tests should display significant positive values. The Japanese white-eye *Zosterops japonicus* (Genbank accession number JN635726) when necessary, and significance of the tests was assessed by 10,000 coalescent simulations on the basis of segregating sites using DNAsp version 5 [28].

Selection also acts on the ratio of non-synonymous to synonymous mutations. When a coding site is under positive selection, it can limit the appearance of other non-synonymous mutations. To identify putative functionally important sites, we performed the McDonald-Kreitmann test [29] using the Japanese white-eye as an outgroup.

We also examined whether significant differentiation occurred between morphs rather than between populations. This is expected if positive selection acts on a cis-regulatory mutation, as a selective sweep is likely to fix distinct haplotypes between morphs. We obtained differentiation indices using an analysis of molecular variance (AMOVA) with morphs as groups as implemented in Arlequin 3.5 [30]. P-values were obtained by performing 10,000 permutations.

## Results

A total of 817 bp of the *MC1R* gene were successfully sequenced. Eight sites were variable, giving a total of nine different haplotypes. Mutations consisted of four non-synonymous and five synonymous substitutions. Non-synonymous substitutions were an Ala^45^→Val^45^, a Val^172^→ Ile^172^ and a Pro^225^→ Ser^225^ for *Z. borbonicus* and an Ala^228^→ Val^228^ for *Z. mauritianus* (Table 3).These amino acids all had a hydrophilic lateral chain except for Proline. Moreover, in chicken Ala^45^ is replaced by a Thr^45^, having a neutral lateral chain, suggesting this site is less constrained. These substitutions do not seem to modify greatly the chemical properties of the protein and are unlikely to have a large impact on the receptoŕs structure. This was supported by McDonald-Kreitman tests which failed to detect any sign of positive selection on amino acid-altering mutations at *MC1R* (Table 4).

**Table 3:** Amino-acids variants observed at the *MC1R* locus in 51 *Z. borbonicus* individuals representing the four Réunion morphs and nine *Z. mauritianus* individuals.

	Variant 1	Variant 2	Variant 3	Variant 4
**Position of the mutation**	C^134^→T^134^	G^514^→A^514^	C^673^→T^673^	C^683^→T^683^
**Amino-acid change**	Ala^45^→Val^45^	Val^172^→ Ile^172^	Pro^225^→ Ser^225^	Ala^228^→ Val^228^
**Brown (lowlands)**	Not found	Heterozygous	Not found	Not found
**Brown (highlands)**	Not found	Heterozygous	Not found	Not found
**Grey**	Not found	Heterozygous	Not found	Not found
**Grey-headed brown**	Heterozygous	Not found	Not found	Not found
**Grey-headed brown-naped brown**	Not found	Heterozygous	Heterozygous	Not found
**Mauritius**	Not found	Not found	Not found	Heterozygous

For each variant the corresponding nucleotide substitution is indicated, with its state (heterozygous or homozygous) in each morph and species studied here. Sequences were numbered in reference to the chicken genome (Genbank accession number: AY220305).

**Table 4:** Results for McDonald-Kreitman neutrality test.

		Non-synonymous mutations		Synonymous mutations	McDonald-Kreitmann test
**Lowland brown**	Fixed		6	3	NS
	Polymorphic		1	1	
**Highland brown**	Fixed		6	2	NS
	Polymorphic		1	3	
**Brown (both lowland and** **highland)**	Fixed		6	2	NS
	Polymorphic		1	3	
**Grey**	Fixed		6	2	NS
	Polymorphic		1	3	
**Grey-headed brown**	Fixed		6	2	NS
	Polymorphic		1	2	
**Grey-headed brown-naped brown**	Fixed		6	2	NS
	Polymorphic		2	3	
**Zosterops borbonicus**	Fixed		6	2	NS
	Polymorphic		3	4	
**Zosterops mauritianus**	Fixed		6	2	NS
	Polymorphic		1	3	

*Zosterops japonicus* sequence was used as an outgroup. NS: non-significant.

**Figure 2:**
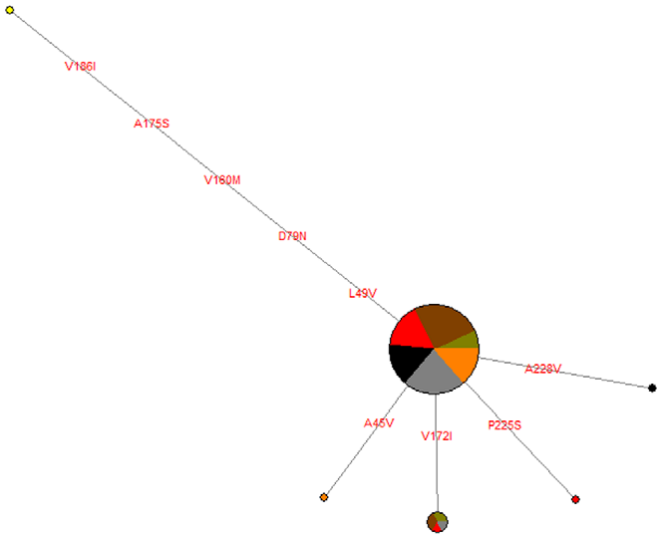
*MC1R* non-synonymous variants network for 120 haplotypes from *Z. borbonicus* and *Z. mauritianus*. This is a median-joining network. Circles represent variants with areas proportional to their sample sizes. Each branch represents a single substitution with amino-acid position indicated. Proportions of individuals of each locality are indicated by pie charts for each haplotype (black: *Z. mauritianus*, light brown: lowland brown morph, dark brown: highland brown morph, grey: grey morph, orange: grey-headed brown morph, red: grey-headed brown-naped brown morph, yellow: outgroup, *Zosterops japonicus*).

No correlation between these substitutions and variation in pigmentation between *Z. borbonicus* morphs was detected (Figure 2). We found several shared mutations between *Z. borbonicus* morphs or between *Z.borbonicus* and *Z. mauritianus*, both synonymous and non-synonymous. Since the Mauritian species is monomorphic across its range, these mutations do not seem to be linked to color variation and might instead represent shared ancestral polymorphism.

Nucleotide diversity was relatively low (π = 0.00078 and 0.00167 for *Z. borbonicus* and *Z. mauritianus* respectively). All neutrality tests were skewed towards negative values in all *Z. borbonicus* morphs (Table 5). However, values were significantly less than zero only for F_s_ and H values in the brown morphs (both lowland and highland populations), the grey morph and the grey-headed brown morph. Negative F_s_ values suggest a role for demographic expansion, whereas negative Fay and Wu’s H could be consistent with long-term purifying selection in explaining patterns of variation at *MC1R* instead of positive selection associated to morphs.

**Table 5:** Diversity statistics and results from selection tests for *Z. borbonicus* morphs.

Morph/Species	π	S	Tajima’s D	Fu and Li’s D*	Fu and Li’s F*	Fu’sF_s_	Fay and Wu’s H
**Z.borbonicus**	0.00078	7	−1.227	−0.561	−0.929	−3.927	−**2.562** *
**Brown**	0.00052	4	−1.304	−1.103	−1.355	−2.658	−**3.300** **
Brown (lowland)	0.00068	2	−0.691	−0.280	−0.423	−0.594	−1.333
Brown (highland)	0.00048	4	−1.574	−0.968	−1.329	−**3.219** *	−**3.356** **
**Grey**	0.00082	4	−0.962	−0.897	−1.060	−1.845	−**2.499** *
**Grey-headed brown**	0.00084	3	−0.708	−0.039	−0.247	−1.098	−**2.367** *
**Grey-headed brown-naped brown**	0.00119	5	−0.946	−0.413	−0.648	−2.344	−1.947

Π: nucleotide diversity. S: number of segregating sites. Significance levels:

* p<0.05;

** p<0.01.

The lack of positive selection associated to morphs was supported by the AMOVA analysis, which did not detect any significant morph effect (φ_ct_ = 0.013, P>0.05). Since no variation in haplotype frequencies was associated to color morphs, no effect of a selective sweep linked to a putative cis-regulatory mutation could be detected.

## Discussion

Despite its frequent involvement in pigmentation patterns in vertebrates, especially in birds (Table 1), *MC1R* does not seem to play a role in explaining variation in plumage pigmentation in *Z. borbonicus*. We found no relationship between plumage pigmentation and variation at the *MC1R* locus, for either synonymous or non-synonymous substitutions, and observed non-synonymous substitutions are unlikely to result in functional changes.

Since we could not sequence the first 23 and last 20 codons of *MC1R* we cannot exclude the possibility that functional modifications occurred in these regions. However, this seems unlikely since the region examined here contains all the sites previously described as important for *MC1R* function in birds [5], [7], [11], [31], [32]. We did not find any of the color-associated mutations already reported in previous studies on birds. Substitutions Val^85^→Met^85^, Glu^92^→Lys^92^ and Asp^119^→Asn^119^ [5], [7], [11] observed in bananaquits (*Coereba flaveola*), snow geese (*Anser caerulescens*) and the chestnut-bellied monarch (*Monarcha castaneiventris*) were not observed here. Similarly, other substitutions like Arg^230^→His^230^ observed in Arctic skuas (*Stercorarius parasiticus*) or Glu^100^→Lys^100^ reported in swans (*Cygnus*) were not found in our study [5], [8]. It is difficult to definitively rule out the possibility that *MC1R* cis-regulatory mutations underlie some pigmentation phenotypes in *Z. borbonicus* or other species. However, in our study, we found no indication for genetic hitchiking in *MC1R* coding sequences, as would be expected if they were linked to positively selected regions in nearby locations.

Our results are instead consistent with those obtained by [11], [14], [15]. Indeed, many studies having shown the involvement of *MC1R* focused on species displaying extreme dimorphism and rarely on variation in patterns of melanin deposition across the body (Table 1). This confirms that *MC1R* is not systematically involved in melanin-based pigmentation changes in birds, reinforcing the notion that understanding the evolution of plumage coloration in species with complex patterns of eumelanin/phaeomelanin deposition requires a wider exploration of other genes within the melanocortin pathway, as well as variation in other candidate genes. Indeed, several genetic and developmental mechanisms are likely to regulate the complex patterns of pigment deposition in feathers [33], [34] possibly interacting with *MC1R* regulatory variation, which also need to be characterized.

A potentially interesting candidate gene that may underlie such mechanisms is *Agouti* (*ASIP*), a paracrine signaling protein antagonist of *MC1R* involved in pigment patterning in domestic quail and chicken [35], [36] and in pocket mice [37]. In addition to *MC1R*, *Agouti* also interacts with *MC3R* and *MC4R* and has pleiotropic effects on food intake, energy expenditure or nociception [2]. Its antagonist, the pro-opiomelanocortin gene (*POMC)*, is also a candidate since it interacts with the entire family of melanocortin receptors (*MCRs*), including *MC1R*, and may play a role in controlling many metabolic functions, such as stress resistance, reproductive investment or immunity [2] [38].

Since mutations in *ASIP* and *POMC* genes appear to be associated with many physiological, behavioral, and life-history traits, not just color, these two genes seem ideal candidates to understand the origin and evolution of complex melanin-based pigmentation polymorphisms. Yet adaptive changes in the pattern formation of eumelanin and phaeomelanin in *Z. borbonicus* and probably many other species are likely to involve a mixture of modifications in the structure and regulation of the genes underlying pigment production, suggesting that mechanisms of plumage color evolution may be more diverse than implied by recent studies of discrete melanic/non melanic polymorphisms.
